# A population-based matched cohort study of early pregnancy outcomes following COVID-19 vaccination and SARS-CoV-2 infection

**DOI:** 10.1038/s41467-022-33937-y

**Published:** 2022-10-17

**Authors:** Clara Calvert, Jade Carruthers, Cheryl Denny, Jack Donaghy, Sam Hillman, Lisa E. M. Hopcroft, Leanne Hopkins, Anna Goulding, Laura Lindsay, Terry McLaughlin, Emily Moore, Jiafeng Pan, Bob Taylor, Fatima Almaghrabi, Bonnie Auyeung, Krishnan Bhaskaran, Cheryl L. Gibbons, Srinivasa Vittal Katikireddi, Colin McCowan, Josie Murray, Maureen O’Leary, Lewis D. Ritchie, Syed Ahmar Shah, Colin R. Simpson, Chris Robertson, Aziz Sheikh, Sarah J. Stock, Rachael Wood

**Affiliations:** 1 Usher Institute, University of Edinburgh, Edinburgh, UK; 2 Faculty of Epidemiology and Population Health, London School of Hygiene and Tropical Medicine, London, UK; 3 Public Health Scotland, Scotland, UK; 4 Bennett Institute for Applied Data Science, Nuffield Department of Primary Care Health Sciences, University of Oxford, Oxford, UK; 5 Department of Mathematics and Statistics, University of Strathclyde, Glasgow, UK; 6 Department of Psychology, School of Philosophy, Psychology and Language Sciences, University of Edinburgh, Edinburgh, UK; 7 MRC/CSO Social & Public Health Sciences Unit, University of Glasgow, Glasgow, UK; 8 School of Medicine, University of St Andrews, St Andrews, UK; 9 Academic Primary Care, University of Aberdeen, Aberdeen, UK; 10 School of Health, Wellington Faculty of Health, Victoria University of Wellington, Wellington, New Zealand

**Keywords:** Viral infection, Epidemiology, SARS-CoV-2

## Abstract

Data on the safety of COVID-19 vaccines in early pregnancy are limited. We conducted a national, population-based, matched cohort study assessing associations between COVID-19 vaccination and miscarriage prior to 20 weeks gestation and, separately, ectopic pregnancy. We identified women in Scotland vaccinated between 6 weeks preconception and 19 weeks 6 days gestation (for miscarriage; n = 18,780) or 2 weeks 6 days gestation (for ectopic; n = 10,570). Matched, unvaccinated women from the pre-pandemic and, separately, pandemic periods were used as controls. Here we show no association between vaccination and miscarriage (adjusted Odds Ratio [aOR], pre-pandemic controls = 1.02, 95% Confidence Interval [CI] = 0.96–1.09) or ectopic pregnancy (aOR = 1.13, 95% CI = 0.92–1.38). We undertook additional analyses examining confirmed SARS-CoV-2 infection as the exposure and similarly found no association with miscarriage or ectopic pregnancy. Our findings support current recommendations that vaccination remains the safest way for pregnant women to protect themselves and their babies from COVID-19.

## Abstract (web summary)

Data on the safety of COVD-19 vaccines in early pregnancy are limited. Here, the authors assess the rates of miscarriage and ectopic pregnancy following vaccination using electronic health record data from Scotland, and find no evidence of increased risks.

## Introduction

COVID-19 vaccination is effective in reducing the risk of severe SARS-CoV-2 infection in pregnant women^1,2^, but high levels of vaccine hesitancy remain in pregnant populations. In England and Scotland, 59.5% and 63.0%, respectively, of women giving birth in January 2022 had received at least one prior dose of COVID-19 vaccination at any point, which was considerably lower than the coverage seen among the general population of reproductive age women at that time^3–5^.

Several factors are likely to contribute to COVID-19 vaccine hesitancy in pregnant women, including exclusion of pregnant women from the initial vaccine trials and hence inconsistent guidance early in the vaccination programme reflecting the lack of safety data at that time^6^. A growing body of evidence suggests that there is no association between COVID-19 vaccination in pregnancy and adverse late pregnancy outcomes, including preterm birth and stillbirth^7^. There is, in contrast, a paucity of data on early pregnancy outcomes. A recent systematic review identified only two studies, conducted in Norway and the US, comparing the risk of miscarriage between vaccinated and unvaccinated women^7^. Neither of these case-control studies found evidence of an association between COVID-19 vaccination and miscarriage. In the Norwegian population-based study utilizing routine records, women with first-trimester miscarriage had lower odds of having had a COVID-19 vaccination in the preceding 5 weeks compared with women with ongoing pregnancies (adjusted odds ratio [aOR] = 0.81, 95% CI = 0.69–0.95), and similar odds of having had a vaccination in the preceding 3 weeks (aOR = 0.91, 95% CI = 0.75–1.10)^8^. In the US study, women with miscarriage had similar odds of having COVID-19 vaccination in the previous 4 weeks compared with women with ongoing pregnancies (aOR = 1.02, 95% CI = 0.96–1.08)^9^. To our knowledge, there are no published data on the relationship between COVID-19 vaccination and ectopic pregnancy, the other major cause of early pregnancy loss.

There is an urgent need for robust evidence on the safety of COVID-19 vaccines in early pregnancy to inform decision-making among women, their healthcare providers, and policymakers. We use population-based pregnancy data from the COVID-19 in Pregnancy in Scotland (COPS) cohort^10,11^ to investigate whether there was any evidence for an association between COVID-19 vaccination between six weeks preconception and up to 19 weeks and 6 days (19 + 6) gestation and miscarriage, and up to 2 weeks and 6 days (2 + 6) and ectopic pregnancy. As a secondary objective, we examined whether there was any evidence for an association between SARS-CoV-2 infection and miscarriage or ectopic pregnancy.

## Results

### Study population

**Fig. 1:**
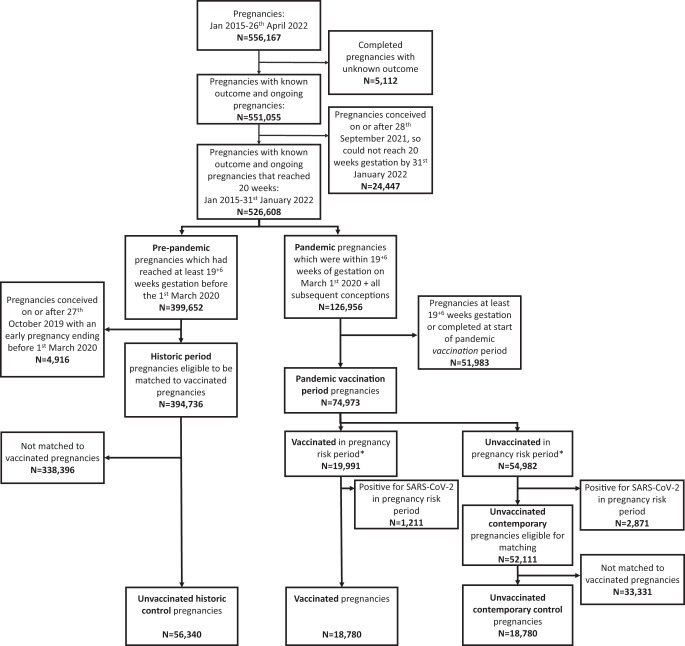
Selection of vaccinated and unvaccinated pregnancy cohorts for miscarriage outcome analysis. Flow diagram showing the selection of pregnancies for the analysis of the association between COVID-19 vaccination and risk of miscarriage. *For miscarriage analysis, vaccination needs to be given between 6 weeks preconception and up to the earliest of (1) end of pregnancy or (2) 19 weeks 6 days gestation.

As of April 26, 2022, the COPS study database contained information on 556,167 pregnancies (in 361,606 women) of which 526,608 were eligible for inclusion: 399,652 from the historical pre-pandemic period and 126,956 from the contemporary pandemic period (Fig. 1).

### Association between COVID-19 vaccination and miscarriage

**Fig. 2:**
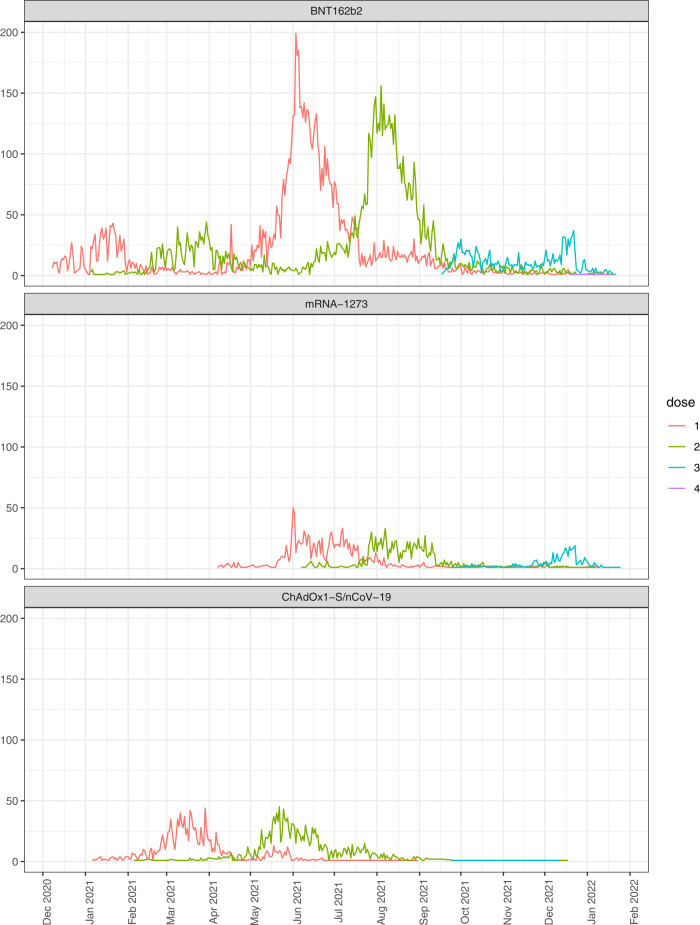
COVID-19 vaccination between 6 weeks preconception and 19 weeks and 6 days gestation over time. Line graph showing the total number of women vaccinated between 6 weeks preconception and up to 19 weeks and 6 days gestation by calendar time, dose number and type of vaccine.

A total of 18,780 pregnant women received COVID-19 vaccination between 6 weeks preconception and 19 + 6 weeks gestation (or the end of pregnancy if earlier) (Fig. 1). The characteristics of the vaccinated cohort, and the matched historical and contemporary control groups, are provided in Table 1. Compared to controls, vaccinated women were more likely to be from the least deprived areas. Pfizer-BioNTech BNT162b2 was the most frequently received vaccine type, and around a quarter of the vaccinated women received two or more doses during the exposure period. Reflecting the roll out of the national vaccination programme in Scotland (Supplementary Fig. 1), most included vaccinations occurred in May to August 2021 (Fig. 2).

**Table 1:** Key characteristics of vaccinated and control groups included in the vaccination and miscarriage analyses

Cohort characteristics		Vaccinated	Unvaccinated (historical)	Unvaccinated (contemporary)
Number of pregnancies		18,780	56,340	18,780
Median age (min–max)		31 (14–51)	31 (14–51)	31 (14–54)
Deprivation (SIMD quintile)	1 (most deprived)	3501 (18.6%)	12,258 (21.8%)	4406 (23.5%)
	2	3503 (18.7%)	11,031 (19.6%)	3803 (20.3%)
	3	3509 (18.7%)	10,018 (17.8%)	3457 (18.4%)
	4	4171 (22.2%)	11,657 (20.7%)	3743 (19.9%)
	5 (least deprived)	3952 (21.0%)	10,807 (19.2%)	3241 (17.3%)
	Unknown	144 (0.8%)	569 (1.0%)	130 (0.7%)
Ethnicity	White	15,897 (84.6%)	37,156 (65.9%)	15,672 (83.5%)
	South Asian	708 (3.8%)	1274 (2.3%)	633 (3.4%)
	Black/Caribbean/African	234 (1.2%)	676 (1.2%)	375 (2.0%)
	Other/mixed ethnicity	596 (3.2%)	1410 (2.5%)	683 (3.6%)
	Unknown	1345 (7.2%)	15,824 (28.1%)	1417 (7.5%)
Urban/rural status	Large urban areas	6801 (36.2%)	21,263 (37.7%)	6999 (37.3%)
	Other urban areas	5974 (31.8%)	20,171 (35.8%)	6561 (34.9%)
	Accessible small towns	1439 (7.7%)	4512 (8.0%)	1390 (7.4%)
	Remote small towns	550 (2.9%)	1597 (2.8%)	572 (3.0%)
	Accessible rural areas	1984 (10.6%)	5611 (10.0%)	2088 (11.1%)
	Remote rural areas	831 (4.4%)	2532 (4.5%)	767 (4.1%)
	Unknown	1201 (6.4%)	654 (1.2%)	403 (2.1%)
Clinical vulnerability	Extremely vulnerable	228 (1.2%)	769 (1.4%)	154 (0.8%)
	Vulnerable	4819 (25.7%)	15,917 (28.3%)	4868 (25.9%)
	Not vulnerable	13,733 (73.1%)	39,654 (70.4%)	13,758 (73.3%)
*Exposure (vaccination)*				
Gestation at first vaccination	Preconception	8816 (46.9%)	–	–
	2 + 0–5 + 6 weeks	4173 (22.2%)	–	–
	6 + 0–10 + 6 weeks	1311 (7.0%)	–	–
	11 + 0–15 + 6 weeks	2468 (13.1%)	–	–
	16 + 0–19 + 6 weeks	2012 (10.7%)	–	–
Number of vaccinations^a^	1	13,792 (73.4%)	–	–
	2+	4988 (26.6%)	–	–
Dose number at first vaccination	Dose 1	10,974 (58.4%)	–	–
	Dose 2	6052 (32.2%)	–	–
	Dose 3	1754 (9.3%)	–	–
Type of vaccination^a^	BNT162b2	13,675 (72.8%)	–	–
	mRNA-1273	2260 (12.0%)	–	–
	ChAdOx1-S/nCoV-19	2612 (13.9%)	–	–
	1+ different type	233 (1.2%)	–	–
*Outcome (miscarriage)*				
Number of miscarriages ≤ 19 + 6 weeks		1716	5566	1878
Gestation at miscarriage	≤10 + 6	1302 (75.9%)	5113 (91.9%)	1313 (69.9%)
	11 + 0–13 + 6	300 (17.5%)	295 (5.3%)	412 (21.9%)
	≥14 + 0	114 (6.6%)	158 (2.8%)	153 (8.1%)
Imputed gestation for miscarriage	Yes	975 (56.8%)	4593 (82.5%)	927 (49.4%)
	No—from ANC booking	576 (33.6%)	308 (5.5%)	721 (38.4%)
	No—from end of pregnancy record	165 (9.6%)	665 (11.9%)	230 (12.2%)
Interval to miscarriage^b^	<2 weeks	84 (4.9%)	223 (4.0%)	101 (5.4%)
	2–5 weeks	258 (15.0%)	657 (11.8%)	262 (14.0%)
	6–9 weeks	536 (31.2%)	1902 (34.2%)	586 (31.2%)
	≥10 weeks	838 (48.8%)	2784 (50.0%)	929 (49.5%)

*SIMD* Scottish Index of multiple deprivation.

^a^Between 6 weeks preconception and the earliest of either: end of pregnancy or 19 + 6 weeks gestation.

^b^Interval between vaccination (or gestation of matching) and miscarriage.

By 19 + 6 weeks gestation, 9.1% (1716/18,780) of pregnancies in the vaccinated cohort ended in miscarriage, compared to 9.9% (5566/56,340) in historical controls and 10.0% (1878/18,780) in contemporary controls (Table 2). Our primary analyses (using historical controls) found no evidence that women vaccinated in pregnancy had higher odds of miscarriage in either the model accounting only for matching factors (OR = 0.98, 95% CI = 0.93–1.04) or adjusted analyses (aOR = 1.02, 95% CI = 0.96–1.09) (Table 2). Results of supplementary analyses (using contemporary controls) were similar (OR = 0.91, 95% CI = 0.85–0.98; aOR = 0.96, 95% CI = 0.88–1.04) (Table 2).

**Table 2:** Odds ratios for the association between COVID-19 vaccination and miscarriage from multinomial logistic regression models

	Number of pregnancies	Number (%) of ongoing pregnancies	Number (%) of miscarriages	Odds ratio (95% CI)^a^	*p*-value	Adjusted odds ratio (95% CI)^b^	*p*-value
**Primary analysis (historical controls)**							
Unvaccinated	56,340	44,669 (79.3%)	5566 (9.9%)	1		1	
Vaccinated	18,780	14,119 (75.2%)	1716 (9.1%)	0.98 (0.93–1.04)	0.55	1.02 (0.96–1.09)	0.49
***BNT162b2 subgroup analysis (historical controls)***							
Unvaccinated	41,025	32,958 (80.3%)	3804 (9.3%)	1		1	
Vaccinated	13,675	10,485 (76.7%)	1147 (8.4%)	0.96 (0.89–1.03)	0.22	1.00 (0.93–1.08)	0.90
***mRNA-1273 subgroup analysis (historical controls)***							
Unvaccinated	6780	5646 (83.3%)	511 (7.5%)	1		1	
Vaccinated	2260	1744 (77.2%)	162 (7.2%)	1.06 (0.88–1.29)	0.52	1.07 (0.87–1.33)	0.51
***ChAdOx1-S/nCoV-19 subgroup analysis (historical controls)***							
Unvaccinated	7836	5564 (71.0%)	1152 (14.7%)	1		1	
Vaccinated	2612	1659 (63.5%)	406 (15.5%)	1.17 (1.03–1.33)	0.01	1.17 (1.03–1.34)	0.02
**Supplementary analysis (contemporary controls)**							
Unvaccinated	18,780	14,162 (75.4%)	1878 (10.0%)	1		1	
Vaccinated	18,780	14,119 (75.2%)	1716 (9.1%)	0.91 (0.85–0.98)	0.01	0.96 (0.88–1.04)	0.35
***BNT162b2 subgroup analysis (contemporary controls)***							
Unvaccinated	13,675	10,494 (76.7%)	1258 (9.2%)	1		1	
Vaccinated	13,675	10,485 (76.7%)	1147 (8.4%)	0.91 (0.83–0.99)	0.03	0.99 (0.89–1.09)	0.81
***mRNA-1273 subgroup analysis (contemporary controls)***							
Unvaccinated	2260	1794 (79.4%)	198 (8.8%)	1		1	
Vaccinated	2260	1744 (77.2%)	162 (7.2%)	0.85 (0.68–1.07)	0.17	1.04 (0.76–1.43)	0.79
***ChAdOx1-S/nCoV-19 subgroup analysis (contemporary controls)***							
Unvaccinated	2612	1731 (66.3%)	389 (14.9%)	1		1	
Vaccinated	2612	1659 (63.5%)	406 (15.5%)	1.08 (0.92–1.27)	0.32	0.92 (0.76–1.11)	0.41

All analyses exclude women with confirmed SARS-CoV-2 infection between 6 weeks preconception and the earliest of either: end of pregnancy or the end of the exposure period at 19 + 6 weeks gestation.

*CI* confidence interval.

^a^Adjusting for matching factors: maternal age and gestational age at the date of vaccination of index vaccinated pregnancy (and season of conception for primary and primary subgroup analysis).

^b^Additionally adjusting for deprivation, urban/rural status, and clinical vulnerability (and ethnicity and season of conception in Supplementary analyses).

Compared to women receiving an mRNA vaccine (Pfizer-BioNTech BNT162b2 or Moderna mRNA-1273), women receiving Oxford/AstraZeneca ChAdOx1-S/nCoV-19 were more likely to be from the most deprived areas, and substantially more likely to categorized as clinically vulnerable or extremely vulnerable (Supplementary Table 1). In subgroup analyses, we found no evidence that women receiving either BNT162b2 or mRNA-1273 vaccines had higher odds of miscarriage compared to historical controls (aOR = 1.00, 95% CI = 0.93–1.08 and aOR = 1.07, 95% CI = 0.87–1.33, respectively) or contemporary controls (aOR = 0.99, 95% CI = 0.89–1.09 and aOR = 1.04, 95% CI = 0.76–1.43, respectively). We did find higher odds of miscarriage among women receiving ChAdOx1-S/nCoV-19 vaccine when compared to historical controls (aOR = 1.17, 95% CI = 1.03–1.34) but not contemporary controls (aOR = 0.92, 95% CI = 0.76–1.11) (Table 2).

### Association between COVID-19 vaccination and ectopic pregnancy

A total of 10,570 pregnant women received COVID-19 vaccination between six weeks preconception and 2 + 6 weeks gestation (Supplementary Fig. 2). Characteristics of the vaccinated cohort, and matched control groups, are provided in Supplementary Table 2. By 19 + 6 weeks gestation, 1.2% of pregnancies (126/10,570) in the vaccinated cohort had ended in ectopic pregnancy, compared to 1.2% of pregnancies (379/31,710) in historical controls and 1.1% (336/31,710) in contemporary controls (Table 3). We found no evidence that women vaccinated in pregnancy had higher odds of ectopic pregnancy in primary or supplementary analyses (aOR = 1.13, 95% CI = 0.92–1.38 and aOR = 1.12, 95% CI = 0.90–1.40 respectively) (Table 3). Similarly, in subgroup analyses, we found no evidence of higher odds of ectopic pregnancy in women receiving any specific vaccine type (Supplementary Table 3 and Table 3).

**Table 3:** Odds ratios for the association between COVID-19 vaccination and ectopic pregnancy from multinomial logistic regression models

	Number of pregnancies	Number (%) of ongoing pregnancies	Number (%) of ectopic pregnancies	Odds ratio (95% CI)^a^	*p*-value	Adjusted odds ratio (95% CI)^b^	*p*-value
**Primary analysis (historical controls)**							
Unvaccinated	31,710	23,438 (73.9%)	379 (1.2%)	1		1	
Vaccinated	10,570	7328 (69.3%)	126 (1.2%)	1.05 (0.86–1.29)	0.61	1.13 (0.92–1.38)	0.25
***BNT162b2 subgroup analysis (historical controls)***							
Unvaccinated	22,020	16,315 (74.1%)	271 (1.2%)	1		1	
Vaccinated	7340	5124 (69.8%)	87 (1.2%)	1.02 (0.80–1.30)	0.90	1.11 (0.87–1.42)	0.39
***mRNA-1273 subgroup analysis (historical controls)***							
Unvaccinated	3120	2405 (77.1%)	22 (0.7%)	1		1	
Vaccinated	1040	705 (67.8%)	13 (1.3%)	1.99 (1.00–3.98)	0.05	2.07 (0.98–4.38)	0.06
***ChAdOx1-S/nCoV-19 subgroup analysis (historical controls)***							
Unvaccinated	5862	4189 (71.5%)	76 (1.3%)	1		1	
Vaccinated	1954	1264 (64.7%)	26 (1.3%)	1.12 (0.72–1.76)	0.61	1.16 (0.74–1.83)	0.52
**Supplementary analysis (contemporary controls)**							
Unvaccinated	31,710	22,901 (72.2%)	336 (1.1%)	1		1	
Vaccinated	10,570	7,328 (69.3%)	126 (1.2%)	1.17 (0.95–1.43)	0.14	1.12 (0.90–1.40)	0.31
***BNT162b2 subgroup analysis (contemporary controls)***							
Unvaccinated	22,020	15,957 (72.5%)	238 (1.1%)	1		1	
Vaccinated	7340	5124 (69.8%)	87 (1.2%)	1.13 (0.89–1.45)	0.32	1.12 (0.86–1.46)	0.41
***mRNA-1273 subgroup analysis (contemporary controls)***							
Unvaccinated	3120	2308 (74.0%)	26 (0.8%)	1		1	
Vaccinated	1040	705 (67.8%)	13 (1.3%)	1.62 (0.83–3.18)	0.16	1.28 (0.55–2.99)	0.57
***ChAdOx1-S/nCoV-19 subgroup analysis (contemporary controls)***							
Unvaccinated	5862	4117 (70.2%)	63 (1.1%)	1		1	
Vaccinated	1954	1264 (64.7%)	26 (1.3%)	1.34 (0.84–2.13)	0.21	1.15 (0.69–1.91)	0.60

All analyses exclude women with confirmed SARS-CoV-2 infection between 6 weeks preconception and the earliest of either: end of pregnancy or the end of the exposure period at 2 + 6 weeks gestation.

*CI* confidence interval.

^a^Adjusting for matching factors: maternal age and gestational age at the date of vaccination of index vaccinated pregnancy (and season of conception for primary and primary subgroup analysis).

^b^Additionally adjusting for deprivation, urban/rural status, and clinical vulnerability (and ethnicity and season of conception in supplementary analyses).

### Association between SARS-CoV-2 infection and miscarriage

A total of 3025 pregnant women had confirmed SARS-CoV-2 infection between 6 weeks preconception and 19 + 6 weeks gestation (Supplementary Fig. 3). Characteristics of this infected cohort, and matched control groups, are provided in Supplementary Table 4. We found no evidence that women infected in pregnancy had higher odds of miscarriage in primary or supplementary analyses (aOR = 1.12, 95% CI = 0.94–1.34 and aOR = 1.00, 95% CI = 0.84–1.20, respectively) (Table 4).

**Table 4:** Odds ratios for the association between SARS-CoV-2 infection and miscarriage from multinomial logistic regression models

	Number of pregnancies	Number (%) of ongoing pregnancies	Number (%) of miscarriages	Odds ratio (95% CI)^a^	*p*-value	Adjusted odds ratio (95% CI)^b^	*p*-value
**Primary analysis (historical controls)**							
Uninfected	9075	7734 (85.2%)	600 (6.6%)	1		1	
Infected	3025	2426 (80.2%)	204 (6.7%)	1.13 (0.95–1.34)	0.16	1.12 (0.94–1.34)	0.22
**Supplementary analysis (contemporary controls)**							
Uninfected	9075	7495 (82.6%)	584 (6.4%)	1		1	
Infected	3025	2426 (80.2%)	204 (6.7%)	1.10 (0.93–1.30)	0.28	1.00 (0.84–1.20)	0.96

All analyses exclude women who received COVID-19 vaccination between 6 weeks preconception and the earliest of either: end of pregnancy or the end of the exposure period at 19 + 6 weeks gestation.

*CI* confidence interval.

^a^Adjusting for matching factors: maternal age and gestational age at the date of infection of index infected pregnancy (and season of conception for primary analysis).

^b^Additionally adjusting for deprivation, urban/rural status, and clinical vulnerability (and ethnicity and season of conception in supplementary analysis).

### Association between SARS-CoV-2 infection and ectopic pregnancy

A total of 915 pregnant women had confirmed SARS-CoV-2 infection between 6 weeks preconception and 2 + 6 weeks gestation (Supplementary Fig. 4). Characteristics of this infected cohort and matched control groups, are provided in Supplementary Table 5. We found no evidence that women infected in pregnancy had higher odds of ectopic pregnancy in primary or supplementary analyses (aOR = 0.76, 95% CI = 0.34–1.69 and aOR = 0.78, 95% CI = 0.34–1.79, respectively) (Table 5).

**Table 5:** Odds ratios for the association between SARS-CoV-2 infection and ectopic pregnancy from multinomial logistic regression models

	Number of pregnancies	Number (%) of ongoing pregnancies	Number (%) of ectopic pregnancies	Odds ratio (95% CI)^a^	*p*-value	Adjusted Odds ratio (95% CI)^b^	*p*-value
**Primary analysis (historical controls)**							
Uninfected	2745	1960 (71.4%)	33 (1.2%)	1		1	
Infected	915	579 (63.3%)	8 (0.9%)	0.80 (0.37–1.75)	0.58	0.76 (0.34–1.69)	0.50
**Supplementary analysis (contemporary controls)**							
Uninfected	2745	1901 (69.3%)	32 (1.2%)	1		1	
Infected	915	579 (63.3%)	8 (0.9%)	0.82 (0.37–1.78)	0.61	0.78 (0.34–1.79)	0.56

All analyses exclude women who received COVID-19 vaccination between 6 weeks preconception and the earliest of either: end of pregnancy or the end of the exposure period at 2 + 6 weeks gestation.

*CI* confidence interval.

^a^Adjusting for matching factors: maternal age and gestational age at the date of infection of index infected pregnancy (and season of conception for primary analysis).

^b^Additionally adjusting for deprivation, urban/rural status, and clinical vulnerability (and ethnicity and season of conception in supplementary analysis).

## Discussion

In our population-based, matched cohort study using routine health data from Scotland, COVID-19 vaccination between 6 weeks preconception to 19 weeks and 6 days gestation was not associated with an increased risk of miscarriage; and COVID-19 vaccination between 6 weeks preconception to 2 weeks and 6 days gestation was not associated with ectopic pregnancy. Results were similar regardless of whether we used historical or contemporary unvaccinated controls. Our results relating to miscarriage are in line with the limited existing evidence including studies that compared the risk in a vaccinated and unvaccinated group with adjustment for key confounders^8,9^, as well as studies that compared the risk in women vaccinated early in pregnancy to previously published historical rates of miscarriage^12,13^. Our results relating to an ectopic pregnancy are novel, as no comparable published evidence is available.

We conducted pre-specified subgroup analyses, exploring the association between specific vaccine types and early pregnancy outcomes. Different vaccine types were offered at different times, and to women in different risk groups, during the roll out of the vaccination programme in Scotland in line with evolving policy (see Supplementary Fig. 1)^14^. The majority of women vaccinated in pregnancy received an mRNA vaccine. We found no association between mRNA vaccines and miscarriage. The ChAdOx1-S/nCoV-19 vaccine was mainly given to women in clinical risk groups (in addition to pregnancy per se, for example, women with pre-existing cardiac or respiratory disease) early in the vaccination programme^15^. We found an association between vaccination with ChAdOx1-S/nCoV-19 and miscarriage when compared with historical controls but not when compared with contemporary controls. Caution is required in the interpretation of these results, particularly those using historical controls. Pregnant women who received ChAdOx1-S/nCoV-19 were much more likely than their matched controls (or women who received an mRNA vaccine) to be classed as clinically vulnerable or clinically extremely vulnerable, reflecting the particular nature of the group receiving this vaccine. Whilst our marker of clinical vulnerability reflects a range of conditions conferring additional vulnerability to severe COVID-19 disease (see Supplementary Table 6), it may not fully account for conditions associated with an increased risk of miscarriage. In addition, regardless of the year of pregnancy, clinical vulnerability status was ascertained from pandemic-era records, which may lead to over-ascertainment of clinical vulnerability in historical controls. This means there is a particular risk of residual confounding affecting the results of the ChAdOx1-S/nCoV-19 subgroup analyses, especially those using historical controls. We found no unusual patterns in the gestation at miscarriage, or the lag between vaccination and miscarriage, for any vaccine types. An analysis of pregnancy outcomes in 121 women who became pregnant whilst participating in clinical trials of ChAdOx1/nCoV-19 found that the rate of miscarriage was no higher in the ChAdOx1/nCoV-19 group than in the control group, with an adjusted risk ratio of 0.84 (95% CI = 0.24–2.90)^16^. The only other study exploring COVID-19 vaccine type and miscarriage that included ChAdOx1-S/nCoV-19, also did not show any evidence of an association between ChAdOx1-S/nCoV-19 and miscarriage (aOR 0.84 in vaccinated women compared to unvaccinated women; 95% CI = 0.48–1.48)^8^. On balance, the evidence relating to ChAdOx1-S/nCoV-19 and miscarriage is reassuring. However, replication of analyses in settings where this vaccine is used more widely in women across different clinical risk profiles would be beneficial.

We found no evidence for an association between SARS-CoV-2 infection and either miscarriage or ectopic pregnancy, but there is imprecision in our estimates due to the relatively small number of women with SARS-CoV-2 infection in the preconception period or early pregnancy. Early in the pandemic, Baud and colleagues reported a case in which a second-trimester miscarriage appeared to be linked with placental infection with SARS-CoV-2^17^, but since then the emerging evidence on infection and miscarriage has been conflicting. Two small hospital-based studies conducted early in the pandemic found no evidence for an association between SARS-CoV-2 infection and the risk of miscarriage^18,19^. More recently, a study from the United Kingdom (UK), in which a sample of women who conceived during the COVID-19 pandemic were asked to self-report if they had a SARS-CoV-2 diagnosis or miscarriage in the first 13 weeks of pregnancy, found an increased risk of miscarriage but with wide confidence intervals and was subject to recall bias (adjusted risk ratio = 1.7, 95% CI = 1.0–3.0)^20^. In our study, we restricted the analysis to only the period when testing was widely available in the community but we cannot rule out that some women that had SARS-CoV-2 infection were misclassified as uninfected if they did not test or report positive test results. The relationship between SARS-CoV-2 infection and miscarriage and ectopic pregnancy warrants further investigation.

The strengths of our study include the use of population-based data covering a whole country and the use of routinely recorded data. Our study design accounted for key biases that tend to impact studies of vaccination in pregnancy, notably immortal time bias and cohort truncation bias^21^. To avoid immortal time bias, we matched on the gestational age at exposure, thereby ensuring that our exposed and control groups were balanced with respect to the underlying risk of early pregnancy outcomes at different gestational ages. We also repeated the analyses using different comparison groups to test the robustness of our results.

There are some limitations that need to be considered. Firstly, we could not include early miscarriages where the woman did not seek healthcare advice. However, as UK guidance is to attend for assessment in any case of suspected pregnancy loss, and we included data from both community and secondary healthcare settings, we anticipate these numbers are small. Secondly, we had to rely on imputed gestation at end of pregnancy for a high proportion of pregnancies ending in early loss, which may have led to misclassification of vaccination or infection status (due to inaccurate assignment of the relevant exposure period) for some pregnancies. Imprecise information on gestation at pregnancy loss is a challenge faced by all studies examining early pregnancy losses, as many losses occur before a dating ultrasound scan is performed and because embryo/foetal demise frequently occurs sometime before the onset of symptoms of pregnancy loss. Thirdly, it was not possible to conduct subgroup analyses by the number of doses when looking at early pregnancy outcomes due to the relatively short exposure window for vaccination. Finally, there remains potential for residual confounding in our analyses. In addition to the issues relating to the measurement of clinical vulnerability discussed above, we were not able to adjust for body mass index (BMI) or smoking; or include diabetes in clinical vulnerability scores, due to substantial differential levels of missing data for these covariates between early pregnancy losses (with high levels of missing data), and those pregnancies where pregnancies continued past the second trimester and a birth record was generated (low levels of missing data).

In conclusion, this study adds robust population-based evidence on the safety of COVID-19 vaccinations for women who are planning to become pregnant or who are in the early stages of pregnancy. Overall, our analyses found no evidence of an increased risk for miscarriage or ectopic pregnancy after COVID-19 vaccination, supporting current recommendations that vaccination remains the safest way for pregnant women to protect themselves and their babies from COVID-19.

## Methods

We conducted a population-based, matched cohort study following a study protocol and statistical analysis plan^22^, and reported results according to the Strengthening the Reporting of Observational Studies in Epidemiology (STROBE) guidelines^23^.

### Study population

Data were obtained from the dynamic, population-based COVID-19 in Pregnancy in Scotland (COPS) cohort^24^, which included all completed and ongoing pregnancies in Scotland from January 1, 2015, onwards. This cohort has been described in detail elsewhere^10,11^. In brief, information on pregnancies, including estimated date of conception and, for completed pregnancies, the pregnancy outcome and gestational age at end of pregnancy were extracted for all women aged 11–55 at conception from antenatal care (ANC) booking records; General Practitioner (GP) records; general acute hospital discharge records (Scottish Morbidity Record (SMR) 01); maternity hospital discharge records (SMR 02); statutory termination of pregnancy notifications (AAS); National Records of Scotland (NRS) statutory live birth registrations; NHS Scotland live birth notifications; and NRS statutory stillbirth registrations. National data on COVID-19 vaccination (including date and type of vaccination) and confirmed SARS-CoV-2 infections were incorporated into the COPS study cohort using unique identifiers.

We used the COPS database as updated on April 26, 2022. We included pregnancies with an estimated conception date up to September 28, 2021, and identified outcomes occurring up to January 31, 2022, hence all included pregnancies could be observed up to 20 weeks gestation (or end of pregnancy if earlier). We excluded the small number of completed pregnancies that had unknown pregnancy outcome (*N* = 5112, 0.9% of all pregnancies included in COPS database).

### COVID-19 vaccination and early pregnancy outcomes

#### Exposure

The COVID-19 vaccination programme started in Scotland on December 8, 2020. A timeline of the programme as relevant to pregnant women is provided in Supplementary Figure 1. Our primary exposure was receipt of a COVID-19 vaccine, including any vaccine type available in Scotland (ChAdOx1-S/nCoV-19 [Oxford/AstraZeneca], BNT162b2 [Pfizer-Biontech], or mRNA-1273 [Moderna]) and any dose (first, second, etc.). Women were included if they were vaccinated from 6 weeks before conception to the end of the outcome-specific exposure period, defined as 19 weeks and 6 days gestation (19 + 6 weeks) for miscarriage and 2 + 6 weeks for ectopic pregnancy. A shorter exposure period was used for ectopic pregnancies as implantation has occurred by 2 + 6 weeks, hence pregnancy location cannot be influenced by subsequent exposures.

#### Outcomes

All pregnancies were categorized as completed or ongoing at 19 + 6 weeks. Completed pregnancies were further categorized by the outcome as miscarriage (including small numbers of molar pregnancies and live births at a non-viable gestational age); ectopic pregnancy; or termination of pregnancy. The outcomes of interest (miscarriage and ectopic pregnancy) were ascertained through ICD-10 or Read Coded Clinical Terms diagnostic codes recorded on hospital discharge or GP records, respectively^25^.

Some source records (notably GP and SMR01 general (non-maternity) hospital discharge records) do not contain information on gestation, and gestation may be missing on other record types. Where gestation at end of pregnancy was unknown, it was assumed to be 10 + 0 weeks for miscarriages and 8 + 0 weeks for ectopic pregnancies. The exception was miscarriages identified through SMR02 maternity hospital discharge records: these records tend to relate to more advanced pregnancies hence 12 + 0 weeks was assumed.

#### Matching to unvaccinated controls

In our primary analyses, we matched vaccinated women to unvaccinated historical (pre-pandemic period) controls. This avoided potential bias from undiagnosed SARS-CoV-2 infection and ensured a sufficient pool of potential controls^26^. Different pregnancies from a single woman could be included in the matched cohort. If a vaccinated woman also had a pregnancy in the pre-pandemic period, that pregnancy was a valid control, as long as the woman was not matched to herself. Potential controls had an estimated conception date from January 1, 2015, up to October 27, 2019, hence all control pregnancies could be observed up to 20 weeks gestation prior to the start of the pandemic on March 1, 2020. Each vaccinated woman was matched to three historical controls by maternal age at conception (±one year), the season of conception (January–March, April–June, July–September, October–December), and gestation (in completed weeks) at first vaccination in pregnancy (e.g. if a woman was vaccinated at 15 weeks gestation, she would be matched to unvaccinated women with an ongoing pregnancy at 15 weeks gestation).

Analyses using historical controls are vulnerable to bias if there are secular trends in the outcomes of interest. We, therefore, conducted supplementary analyses matching vaccinated women to unvaccinated contemporary (pandemic vaccination period) controls. Controls were considered unvaccinated if they did not receive any COVID-19 vaccination between 6 weeks of preconception and the end of the outcome-specific exposure period. Vaccinated women were matched to contemporary controls by maternal age at conception (±1 year, except at tail ends of distribution with women aged <20 or >40 matched within these age groups) and gestation (in completed weeks) at first vaccination in pregnancy. Each vaccinated woman was matched to one control in the miscarriage analysis (due to a limited pool of controls), and three controls in the ectopic analysis.

As our aim was to assess the safety of vaccination per se (rather than identifying any impact of vaccination on pregnancy outcomes mediated through reduced risk of SARS-CoV-2 infection), in all analyses we excluded pregnancies if the woman had confirmed SARS-CoV-2 infection between 6 weeks preconception and the earliest of: (1) the end of pregnancy or (2) the end of the outcome-specific exposure period. This exclusion was applied before the matched cohorts were selected.

### SARS-CoV-2 infection and early pregnancy outcomes

We also compared early pregnancy outcomes in women with and without confirmed SARS-CoV-2 infection. Here our exposure of interest was confirmed SARS-CoV-2 infection from 6 weeks preconception to the end of the outcome-specific exposure period, between May 18, 2020 (the date from which widespread community viral testing was available in Scotland) and January 31, 2022. Confirmed infection was defined in line with national guidance^27^. Up to January 5, 2022, this was by positive SARS-CoV-2 reverse transcription (RT) polymerase chain reaction (PCR) test result. From January 6, 2022, by a positive SARS-CoV-2 RT-PCR test result or by a positive lateral flow device (LFD) test result (unless the positive LFD result was followed by a negative RT-PCR result within 48 h). The date of onset of SARS-CoV-2 was defined as the date the woman’s first positive sample was taken. Subsequent positive test results within 90 days of an index positive result were discounted, with any positive test taken >90 days following the first positive sample considered a second or subsequent infection. Taking a similar approach to the vaccination analysis, we matched infected women to uninfected historical controls on maternal age, the season of conception, and gestational age at infection for primary analyses and to uninfected contemporary controls on maternal age and gestational age at infection for supplementary analyses (with 3:1 matching possible for all groups due to sufficient controls). Historical controls were used for the primary analysis to avoid any misclassification of women with undiagnosed infection as uninfected. In all infection analyses, we excluded women who received COVID-19 vaccination between 6 weeks preconception and the earliest of (1) the end of pregnancy or (2) the end of the outcome-specific exposure period.

### Statistical analyses

We undertook descriptive analyses of the characteristics of exposed and control groups; details of exposures; and pregnancy outcomes by 19 + 6 weeks gestation (see Supplementary Table 6 for details of available characteristics/confounder variables). Multinomial logistic regression was used to estimate the odds of each early pregnancy outcome (i.e. miscarriage, ectopic pregnancy, and termination) compared to ongoing pregnancy by 19 + 6 weeks gestation among vaccinated compared to unvaccinated pregnant women. A multinomial logistic regression allowed us to jointly model all the potential early pregnancy outcomes, rather than restricting to only specific outcomes post-exposure, which would have implications for the balance achieved by matching. Matching characteristics (maternal age, gestational age, and, in primary analyses only, the season of conception) were included in all models. Additional potential confounders (maternal deprivation level, urban/rural residence status, clinical vulnerability, and in supplementary analyses only, ethnicity and season of conception) were included in models providing adjusted odds ratios (aORs). In these analyses, we included a separate unknown/missing group for confounders where there were low levels of missing data, as well as conducting additional analyses removing individuals with missing data from models to assess whether the inclusion of these data were impacting our results. Ethnicity was not included in primary analyses due to the high level of missing data for historical controls. Other potential confounders of interest, specifically smoking status, body mass index, and presence of diabetes was not included in any analyses, due to high levels of missing data on these variables for women whose pregnancies ended in an early loss. The same analytical process was followed to assess the association between vaccination at up to 2 + 6 weeks gestation and ectopic pregnancy and between SARS-CoV-2 infection and each early pregnancy outcome.

Analyses were conducted in R 3.6.1.

### Subgroup analyses

We undertook planned subgroup analyses stratifying our primary and supplemental vaccination analyses by type of vaccine (ChAdOx1-S/nCoV-19, BNT162b2, or mRNA-1273). We excluded pregnant women (and their matched controls) from these subgroup analyses if different vaccine types were received in the exposure period.

### Reporting summary

Further information on research design is available in the Nature Research Reporting Summary linked to this article.

## Supplementary information

Supplementary Information

Peer Review File

Reporting Summary

## Data Availability

Aggregate data files on COVID-19 vaccinations and SARS-CoV-2 infections among pregnant women are available here: https://www.opendata.nhs.scot/organization/health_protection. Patient-level data underlying this article cannot be shared publicly due to data protection and confidentiality requirements. Public Health Scotland is the data holder for the data used in this study. Data can be made available to approved researchers for analysis after securing relevant permissions from the data holders via the Public Benefit and Privacy Panel. Enquiries regarding data availability should be directed to phs.edris@phs.scot.

## Code availability

Metadata and code are available on GitHub at https://github.com/Public-Health-Scotland/COPS-public^28^.
