# Supplementary material for: A population-based matched cohort study of early pregnancy outcomes following COVID-19 vaccination and SARS-CoV-2 infection
Source: Nat Commun. 2022 Oct 17;13:6124. doi: 10.1038/s41467-022-33937-y (PMC9574832; doi:10.1038/s41467-022-33937-y)
Supplement: Supplementary file 1 — Supplementary Information [file 41467_2022_33937_MOESM1_ESM.pdf]

Supplementary Information

A population-based matched cohort study of early pregnancy outcomes following COVID-19 vaccination and SARS-CoV-2 infection

Supplementary Table 1: Key characteristics of vaccinated and control groups included in the vaccination and miscarriage subgroup analyses

	BNT162b2 sub-group analysis			mRNA-1273 sub-group analysis			ChAdOx1-S/nCoV-19 subgroup analysis		
	**Vaccinated cohort**	**Unvaccinated historical controls**	**Unvaccinated contemporary controls**	**Vaccinated cohort**	**Unvaccinated historical controls**	**Unvaccinated contemporary controls**	**Vaccinated cohort**	**Unvaccinated historical controls**	**Unvaccinated contemporary controls**
**Key characteristics**									
**Number of pregnancies**	13675	41025	13675	2260	6780	2260	2612	7836	2612
**Median age (min-max)**	31 (14-49)	31 (14-50)	31 (14-52)	31 (17-51)	31 (16-51)	31 (14-46)	32 (17-50)	32 (16-51)	32 (14-54)
**Deprivation (SIMD quintile)**									
1 (most deprived)	2361 (17.3%)	8986 (21.9%)	3216 (23.5%)	461 (20.4%)	1459 (21.5%)	523 (23.1%)	649 (24.8%)	1673 (21.4%)	611 (23.4%)
2	2523 (18.4%)	8039 (19.6%)	2821 (20.6%)	412 (18.2%)	1325 (19.5%)	444 (19.6%)	539 (20.6%)	1544 (19.7%)	498 (19.1%)
3	2667 (19.5%)	7325 (17.9%)	2515 (18.4%)	315 (13.9%)	1181 (17.4%)	405 (17.9%)	480 (18.4%)	1384 (17.7%)	498 (19.1%)
4	3168 (23.2%)	8499 (20.7%)	2717 (19.9%)	459 (20.3%)	1436 (21.2%)	471 (20.8%)	487 (18.6%)	1585 (20.2%)	499 (19.1%)
5 (least deprived)	2850 (20.8%)	7782 (19.0%)	2319 (17.0%)	599 (26.5%)	1329 (19.6%)	406 (18.0%)	434 (16.6%)	1531 (19.5%)	478 (18.3%)
Unknown	106 (0.8%)	394 (1.0%)	87 (0.6%)	14 (0.6%)	50 (0.7%)	11 (0.5%)	23 (0.9%)	119 (1.5%)	28 (1.1%)
**Ethnicity**									
White	11639 (85.1%)	27297 (66.5%)	11455 (83.8%)	1746 (77.3%)	4608 (68.0%)	1891 (83.7%)	2314 (88.6%)	4829 (61.6%)	2131 (81.6%)
South Asian	496 (3.6%)	964 (2.3%)	458 (3.3%)	123 (5.4%)	140 (2.1%)	82 (3.6%)	76 (2.9%)	149 (1.9%)	88 (3.4%)
Black/Caribbean /African	133 (1.0%)	473 (1.2%)	266 (1.9%)	61 (2.7%)	81 (1.2%)	46 (2.0%)	40 (1.5%)	113 (1.4%)	58 (2.2%)
Other/mixed ethnicity	413 (3.0%)	1022 (2.5%)	491 (3.6%)	115 (5.1%)	168 (2.5%)	88 (3.9%)	59 (2.3%)	199 (2.5%)	99 (3.8%)
Unknown	994 (7.3%)	11269 (27.5%)	1005 (7.3%)	215 (9.5%)	1783 (26.3%)	153 (6.8%)	123 (4.7%)	2546 (32.5%)	236 (9.0%)
**Urban/rural status**									
Large urban areas	4255 (31.1%)	15503 (37.8%)	5113 (37.4%)	1504 (66.5%)	2493 (36.8%)	843 (37.3%)	904 (34.6%)	2985 (38.1%)	962 (36.8%)
Other urban areas	4686 (34.3%)	14658 (35.7%)	4783 (35.0%)	316 (14.0%)	2529 (37.3%)	776 (34.3%)	938 (35.9%)	2744 (35.0%)	926 (35.5%)
Accessible small towns	1103 (8.1%)	3310 (8.1%)	1026 (7.5%)	97 (4.3%)	528 (7.8%)	157 (6.9%)	229 (8.8%)	624 (8.0%)	193 (7.4%)
Remote small towns	442 (3.2%)	1155 (2.8%)	414 (3.0%)	16 (0.7%)	184 (2.7%)	72 (3.2%)	89 (3.4%)	245 (3.1%)	71 (2.7%)
Accessible rural areas	1568 (11.5%)	4084 (10.0%)	1479 (10.8%)	131 (5.8%)	675 (10.0%)	290 (12.8%)	270 (10.3%)	772 (9.9%)	286 (10.9%)
Remote rural areas	696 (5.1%)	1858 (4.5%)	572 (4.2%)	17 (0.8%)	310 (4.6%)	84 (3.7%)	116 (4.4%)	336 (4.3%)	102 (3.9%)
Unknown	925 (6.8%)	457 (1.1%)	288 (2.1%)	179 (7.9%)	61 (0.9%)	38 (1.7%)	66 (2.5%)	130 (1.7%)	72 (2.8%)

**Clinical vulnerability**									
Extremely vulnerable	109 (0.8%)	570 (1.4%)	110 (0.8%)	14 (0.6%)	82 (1.2%)	16 (0.7%)	104 (4.0%)	111 (1.4%)	26 (1.0%)
Vulnerable	3261 (23.8%)	11709 (28.5%)	3542 (25.9%)	500 (22.1%)	1878 (27.7%)	571 (25.3%)	1013 (38.8%)	2169 (27.7%)	685 (26.2%)
Not vulnerable	10305 (75.4%)	28746 (70.1%)	10023 (73.3%)	1746 (77.3%)	4820 (71.1%)	1673 (74.0%)	1495 (57.2%)	5556 (70.9%)	1901 (72.8%)
**Exposure (vaccination)**									
**Gestation at first vaccination within exposure period**									
Preconception	6126 (44.8%)	-	-	828 (36.6%)	-	-	1653 (63.3%)	-	-
2+0-5+6 weeks	2975 (21.8%)	-	-	486 (21.5%)	-	-	691 (26.5%)	-	-
6+0-10+6 weeks	1001 (7.3%)	-	-	166 (7.3%)	-	-	141 (5.4%)	-	-
11+0-15+6 weeks	1978 (14.5%)	-	-	420 (18.6%)	-	-	70 (2.7%)	-	-
16+0-19+6 weeks	1595 (11.7%)	-	-	360 (15.9%)	-	-	57 (2.2%)	-	-
**Number of vaccinations***									
1	10025 (73.3%)	-	-	1615 (71.5%)	-	-	2152 (82.4%)	-	-
2+	3650 (26.7%)	-	-	645 (28.5%)	-	-	460 (17.6%)	-	-
**Dose number at first vaccination within exposure period**									
Dose 1	8181 (59.8%)	-	-	1367 (60.5%)	-	-	1394 (53.4%)	-	-
Dose 2	4123 (30.1%)	-	-	514 (22.7%)	-	-	1215 (46.5%)	-	-
Dose 3	1371 (10%)	-	-	379 (16.8%)	-	-	3 (0.1%)	-	-
**Outcome (miscarriage)**									
**Pregnancies ending in miscarriage up to 19+6 weeks**	1147	3804	1258	162	511	198	406	1152	389
**Gestation at miscarriage**									
<=10+6	852 (74.3%)	3467 (91.1%)	867 (68.9%)	127 (78.4%)	468 (91.6%)	133 (67.2%)	322 (79.3%)	1082 (93.9%)	290 (74.6%)
11+0-13+6	212 (18.5%)	223 (5.9%)	285 (22.7%)	26 (16.0%)	25 (4.9%)	46 (23.2%)	62 (15.3%)	44 (3.8%)	74 (19.0%)
>=14+0	83 (7.2%)	114 (3.0%)	106 (8.4%)	9 (5.6%)	18 (3.5%)	19 (9.6%)	22 (5.4%)	26 (2.3%)	25 (6.4%)

**Imputed gestation for miscarriage**									
Yes	638 (55.6%)	3108 (81.7%)	619 (49.2%)	103 (63.6%)	426 (83.4%)	87 (43.9%)	233 (57.4%)	967 (83.9%)	207 (53.2%)
No – available from ANC booking	394 (34.4%)	224 (5.9%)	484 (38.5%)	50 (30.9%)	29 (5.7%)	87 (43.9%)	132 (32.5%)	52 (4.5%)	138 (35.5%)
No – available from end of pregnancy record	115 (10.0%)	472 (12.4%)	155 (12.3%)	9 (5.6%)	56 (11.0%)	24 (12.1%)	41 (10.1%)	133 (11.5%)	44 (11.3%)
**Lag between first vaccination (or date of matching in unvaccinated) and miscarriage**									
<2 weeks	56 (4.9%)	158 (4.2%)	76 (6.0%)	11 (6.8%)	38 (7.4%)	18 (9.1%)	17 (4.2%)	27 (2.3%)	7 (1.8%)
2-5 weeks	166 (14.5%)	483 (12.7%)	180 (14.3%)	33 (20.4%)	72 (14.1%)	33 (16.7%)	59 (14.5%)	101 (8.8%)	48 (12.3%)
6-9 weeks	357 (31.1%)	1299 (34.1%)	391 (31.1%)	48 (29.6%)	180 (35.2%)	58 (29.3%)	130 (32.0%)	408 (35.4%)	132 (33.9%)
>=10 weeks	568 (49.5%)	1864 (49.0%)	611 (48.6%)	70 (43.2%)	221 (43.2%)	89 (44.9%)	200 (49.3%)	616 (53.5%)	202 (51.9%)

SIMD=Scottish Index of Multiple Deprivation

*Between 6 weeks pre-conception and the earliest of either: end of pregnancy or 19+6 weeks gestation

All groups exclude women with confirmed SARS-CoV-2 infection between 6 weeks preconception and the earliest of either: end of pregnancy or the end of the exposure period at 2+6 weeks gestation

Supplementary Table 2: Key characteristics of vaccinated and control groups included in the vaccination and ectopic pregnancy analyses

	Vaccinated cohort	Unvaccinated historical controls	Unvaccinated contemporary controls
**Key characteristics**			
**Number of pregnancies**	10,570	31,710	31,710
**Median age (min-max)**	31 (15-51)	31 (14-52)	31 (12-54)
**Deprivation (SIMD quintile)**			
1 (most deprived)	2,147 (20.3%)	7,034 (22.2%)	7,351 (23.2%)
2	2,101 (19.9%)	6,378 (20.1%)	6,520 (20.6%)
3	1,941 (18.4%)	5,606 (17.7%)	5,763 (18.2%)
4	2,286 (21.6%)	6,448 (20.3%)	6,359 (20.1%)
5 (least deprived)	2,000 (18.9%)	5,860 (18.5%)	5,477 (17.3%)
Unknown	95 (0.9%)	384 (1.2%)	240 (0.8%)
**Ethnicity**			
White	8,873 (83.9%)	20,243 (63.8%)	26,465 (83.5%)
South Asian	367 (3.5%)	735 (2.3%)	1,071 (3.4%)
Black/Caribbean /African	121 (1.1%)	345 (1.1%)	572 (1.8%)
Other/mixed ethnicity	298 (2.8%)	776 (2.4%)	1,125 (3.5%)
Unknown	911 (8.6%)	9,611 (30.3%)	2,477 (7.8%)
**Urban/rural status**			
Large urban areas	3,769 (35.7%)	12,124 (38.2%)	11,894 (37.5%)
Other urban areas	3,452 (32.7%)	11,151 (35.2%)	11,144 (35.1%)
Accessible small towns	773 (7.3%)	2,508 (7.9%)	2,345 (7.4%)
Remote small towns	323 (3.1%)	936 (3.0%)	925 (2.9%)
Accessible rural areas	1,046 (9.9%)	3,159 (10.0%)	3,371 (10.6%)
Remote rural areas	444 (4.2%)	1,396 (4.4%)	1,331 (4.2%)
Unknown	763 (7.2%)	436 (1.4%)	700 (2.2%)
**Clinical vulnerability**			
Extremely vulnerable	120 (1.1%)	465 (1.5%)	275 (0.9%)
Vulnerable	2,760 (26.1%)	8,959 (28.3%)	8,231 (26.0%)
Not vulnerable	7,690 (72.8%)	22,286 (70.3%)	23,204 (73.2%)
**Exposure (vaccination)**			
**Gestation at first vaccination within exposure period**			
Preconception	9,291 (87.9%)	-	-
2 weeks (2+0 to 2+6)	1,279 (12.1%)	-	-
**Number of vaccinations***			
1	10,539 (99.7%)	-	-
2+	31 (0.3%)	-	-
**Dose number at first vaccination within exposure period**			
Dose 1	5,719 (54.1%)	-	-
Dose 2	4,832 (45.7%)	-	-
Dose 3	19 (0.2%)	-	-

**Type of vaccination****			
BNT162b2	7,340 (69.4%)	-	-
mRNA-1273	1,040 (9.4%)	-	-
ChAdOx1-S/nCoV-19	1,954 (18.5%)	-	-
1+ different type	236 (2.2%)	-	-
**Outcome (ectopic pregnancy)**			
**Pregnancies ending in ectopic pregnancy up to 19+6 weeks**	126	379	336
**Gestation at ectopic pregnancy**			
<=10+6	125 (99.2%)	376 (99.2%)	324 (96.4%)
11+0-13+6	1 (0.8%)	2 (0.5%)	8 (2.4%)
>=14+0	0	1 (0.3%)	4 (1.2%)
**Imputed gestation for ectopic pregnancy**			
Yes	106 (84.1%)	334 (88.1%)	270 (80.4%)
No – available from ANC booking	7 (5.6%)	3 (0.8%)	32 (9.5%)
No – available from end of pregnancy record	13 (10.3%)	42 (11.1%)	34 (10.1%)
**Lag between first vaccination (or gestational week of matching in unvaccinated) and ectopic pregnancy**			
<=5 weeks	2 (1.6%)	9 (2.4%)	14 (4.2%)
6-9 weeks	65 (51.6%)	219 (57.8%)	182 (54.2%)
>=10 weeks	59 (46.8%)	151 (39.8%)	140 (41.7%)

SIMD=Scottish Index of Multiple Deprivation
*Between 6 weeks preconception and the earliest of either: end of pregnancy or 2+6 weeks gestation
All groups exclude women with confirmed SARS-CoV-2 infection between 6 weeks preconception and the earliest of either: end of pregnancy or the end of the exposure period at 2+6 weeks gestation

Supplementary Table 3: Key characteristics of vaccinated and control groups included in the vaccination and ectopic pregnancy subgroup analyses

	BNT162b2 sub-group analysis			mRNA-1273 sub-group analysis			ChAdOx1-S/nCoV-19 subgroup analysis		
	**Vaccinated cohort**	**Unvaccinated historical controls**	**Unvaccinated contemporary controls**	**Vaccinated cohort**	**Unvaccinated historical controls**	**Unvaccinated contemporary controls**	**Vaccinated cohort**	**Unvaccinated historical controls**	**Unvaccinated contemporary controls**
**Key characteristics**									
**Number of pregnancies**	7340	22020	22020	1040	3120	3120	1954	5862	5862
**Median age (min-max)**	30 (15-49)	30 (14-50)	30 (12-53)	30 (18-51)	30 (17-52)	30 (14-48)	32 (18-50)	32 (17-51)	32 (14-54)
**Deprivation (SIMD quintile)**									
1 (most deprived)	1379 (18.8%)	4950 (22.5%)	5140 (23.3%)	250 (24.0%)	729 (23.4%)	769 (24.6%)	492 (25.2%)	1244 (21.2%)	1293 (22.1%)
2	1458 (19.9%)	4461 (20.3%)	4597 (20.9%)	208 (20.0%)	631 (20.2%)	621 (19.9%)	404 (20.7%)	1156 (19.7%)	1167 (19.9%)
3	1402 (19.1%)	3889 (17.7%)	4000 (18.2%)	138 (13.3%)	521 (16.7%)	576 (18.5%)	350 (17.9%)	1068 (18.2%)	1040 (17.7%)
4	1664 (22.7%)	4442 (20.2%)	4395 (20.0%)	201 (19.3%)	641 (20.5%)	625 (20.0%)	363 (18.6%)	1196 (20.4%)	1210 (20.6%)
5 (least deprived)	1374 (18.7%)	4016 (18.2%)	3728 (16.9%)	234 (22.5%)	568 (18.2%)	507 (16.2%)	323 (16.5%)	1117 (19.1%)	1103 (18.8%)
Unknown	63 (0.9%)	262 (1.2%)	160 (0.7%)	9 (0.9%)	30 (1.0%)	22 (0.7%)	22 (1.1%)	81 (1.4%)	49 (0.8%)
**Ethnicity**									
White	6189 (84.3%)	14097 (64.0%)	18468 (83.9%)	750 (72.1%)	2078 (66.6%)	2627 (84.2%)	1730 (88.5%)	3618 (61.7%)	4812 (82.1%)
South Asian	239 (3.3%)	509 (2.3%)	728 (3.3%)	63 (6.1%)	66 (2.1%)	94 (3.0%)	53 (2.7%)	139 (2.4%)	216 (3.7%)
Black/Caribbean /African	64 (0.9%)	217 (1.0%)	375 (1.7%)	29 (2.8%)	34 (1.1%)	58 (1.9%)	28 (1.4%)	73 (1.2%)	116 (2.0%)
Other/mixed ethnicity	187 (2.5%)	521 (2.4%)	748 (3.4%)	56 (5.4%)	83 (2.7%)	104 (3.3%)	47 (2.4%)	152 (2.6%)	239 (4.1%)
Unknown	661 (9.0%)	6676 (30.3%)	1701 (7.7%)	142 (13.7%)	859 (27.5%)	237 (7.6%)	96 (4.9%)	1880 (32.1%)	479 (8.2%)
**Urban/rural status**									
Large urban areas	2238 (30.5%)	8346 (37.9%)	8198 (37.2%)	713 (68.6%)	1194 (38.3%)	1151 (36.9%)	679 (34.7%)	2310 (39.4%)	2245 (38.3%)
Other urban areas	2574 (35.1%)	7788 (35.4%)	7816 (35.5%)	135 (13.0%)	1149 (36.8%)	1079 (34.6%)	710 (36.3%)	1997 (34.1%)	2009 (34.3%)
Accessible small towns	555 (7.6%)	1739 (7.9%)	1623 (7.4%)	40 (3.8%)	255 (8.2%)	254 (8.1%)	167 (8.5%)	464 (7.9%)	429 (7.3%)
Remote small towns	249 (3.4%)	683 (3.1%)	657 (3.0%)	6 (0.6%)	78 (2.5%)	98 (3.1%)	65 (3.3%)	157 (2.7%)	152 (2.6%)
Accessible rural areas	788 (10.7%)	2179 (9.9%)	2332 (10.6%)	49 (4.7%)	285 (9.1%)	337 (10.8%)	191 (9.8%)	599 (10.2%)	640 (10.9%)

Remote rural areas	357 (4.9%)	983 (4.5%)	922 (4.2%)	3 (0.3%)	125 (4.0%)	131 (4.2%)	82 (4.2%)	248 (4.2%)	244 (4.2%)
Unknown	579 (7.9%)	302 (1.4%)	472 (2.1%)	94 (9.0%)	34 (1.1%)	70 (2.2%)	60 (3.1%)	87 (1.5%)	143 (2.4%)
**Clinical vulnerability**									
Extremely vulnerable	48 (0.7%)	317 (1.4%)	193 (0.9%)	0 (0.0%)	42 (1.3%)	25 (0.8%)	72 (3.7%)	94 (1.6%)	51 (0.9%)
Vulnerable	1754 (23.9%)	6165 (28.0%)	5675 (25.8%)	207 (19.9%)	914 (29.3%)	832 (26.7%)	751 (38.4%)	1676 (28.6%)	1539 (26.3%)
Not vulnerable	5538 (75.4%)	15538 (70.6%)	16152 (73.4%)	833 (80.1%)	2164 (69.4%)	2263 (72.5%)	1131 (57.9%)	4092 (69.8%)	4272 (72.9%)
**Exposure (vaccination)**									
**Gestation at first vaccination within exposure period**									
Preconception	6434 (87.7%)	-	-	891 (85.7%)	-	-	1741 (89.1%)	-	-
2 weeks (2+0 to 2+6)	906 (12.3%)	-	-	149 (14.3%)	-	-	213 (10.9%)	-	-
**Number of vaccinations***		-	-		-	-		-	-
1	7314 (99.6%)	-	-	1039 (99.9%)	-	-	1950 (99.8%)	-	-
2+	26 (0.4%)	-	-	1 (0.1%)	-	-	4 (0.2%)	-	-
**Dose number at first vaccination within exposure period**									
Dose 1	4064 (55.4%)	-	-	626 (60.2%)	-	-	1002 (51.3%)	-	-
Dose 2	3258 (44.4%)	-	-	414 (39.8%)	-	-	951 (48.7%)	-	-
Dose 3	18 (0.2%)			0			1 (0.1%)		
**Outcome (ectopic pregnancy)**									
**Pregnancies ending in ectopic pregnancy up to 19+6 weeks**	87	271	238	13	22	26	26	76	63
**Gestation at ectopic pregnancy**									
<=10+6	86 (98.9%)	268 (98.9%)	228 (95.8%)	13 (100%)	22 (100%)	25 (96.2%)	26 (100%)	76 (100%)	62 (98.4%)
11+0-13+6	1 (1.1%)	2 (0.7%)	7 (2.9%)	0	0	0	0	0	1 (1.6%)

>=14+0	0	1 (0.4%)	3 (1.3%)	0	0	1 (3.8%)	0	0	0
**Imputed gestation for ectopic pregnancy**									
Yes	71 (81.6%)	237 (87.5%)	188 (79.0%)	13 (100%)	19 (86.4%)	21 (80.8%)	22 (84.6%)	70 (92.1%)	52 (82.5%)
No – available from ANC booking	5 (5.7%)	3 (1.1%)	24 (10.1%)	0	0	4 (15.4%)	2 (7.7%)	0	4 (6.3%)
No – available from end of pregnancy record	11 (12.6%)	31 (11.4%)	26 (10.9%)	0	3 (13.6%)	1 (3.8%)	2 (7.7%)	6 (7.9%)	7 (11.1%)
**Lag between first vaccination (or gestational week of matching in unvaccinated) and ectopic pregnancy**									
<=5 weeks	2 (2.3%)	6 (2.2%)	11 (4.6%)	0	1 (4.5%)	1 (3.8%)	0	2 (2.6%)	2 (3.2%)
6-9 weeks	45 (51.7%)	166 (61.3%)	132 (55.5%)	10 (76.9%)	10 (45.5%)	15 (57.7%)	10 (38.5%)	38 (50.0%)	33 (52.4%)
>=10 weeks	40 (46.0%)	99 (36.5%)	95 (39.9%)	3 (23.1%)	11 (50.0%)	10 (38.5%)	16 (61.5%)	36 (47.4%)	28 (44.4%)

SIMD=Scottish Index of Multiple Deprivation

**Between 6 weeks preconception and the earliest of either: end of pregnancy or 2+6 weeks gestation

All groups exclude women with confirmed SARS-CoV-2 infection between 6 weeks preconception and the earliest of either: end of pregnancy or the end of the exposure period at 2+6 weeks gestation

Supplementary Table 4: Key characteristics of infected and control groups included in the infection and miscarriage analyses

	**Infected cohort**	**Uninfected historical controls**	**Uninfected contemporary controls**
**Key characteristics**			
**Number of pregnancies**	3,025	9,075	9,075
**Median age (min-max)**	28 (14-48)	28 (14-48)	28 (13-51)
**Deprivation (SIMD quintile)**			
1 (most deprived)	938 (31.0%)	2,195 (24.2%)	2,425 (26.7%)
2	742 (24.5%)	1,907 (21.0%)	1,940 (21.4%)
3	487 (16.1%)	1,690 (18.6%)	1,625 (17.9%)
4	481 (15.9%)	1,691 (18.6%)	1,692 (18.6%)
5 (least deprived)	363 (12.0%)	1,526 (16.8%)	1,347 (14.8%)
Unknown	14 (0.5%)	66 (0.7%)	46 (0.5%)
**Ethnicity**			
White	2,679 (88.6%)	6,314 (69.6%)	7,642 (84.2%)
South Asian	100 (3.3%)	222 (2.4%)	270 (3.0%)
Black/Caribbean /African	40 (1.3%)	97 (1.1%)	145 (1.6%)
Other/mixed ethnicity	78 (2.6%)	214 (2.4%)	330 (3.6%)
Unknown	128 (4.2%)	2,228 (24.6%)	688 (7.6%)
**Urban/rural status**			
Large urban areas	1,247 (41.2%)	3,270 (36.0%)	3,255 (35.9%)
Other urban areas	1,077 (35.6%)	3,481 (38.4%)	3,429 (37.8%)
Accessible small towns	196 (6.5%)	722 (8.0%)	659 (7.3%)
Remote small towns	53 (1.8%)	283 (3.1%)	277 (3.1%)
Accessible rural areas	214 (7.1%)	859 (9.5%)	941 (10.4%)
Remote rural areas	75 (2.5%)	385 (4.2%)	359 (4.0%)
Unknown	163 (5.4%)	75 (0.8%)	155 (1.7%)
**Clinical vulnerability**			
Extremely vulnerable	18 (0.6%)	114 (1.3%)	70 (0.8%)
Vulnerable	812 (26.8%)	2,543 (28.0%)	2,393 (26.4%)
Not vulnerable	2,195 (72.6%)	6,418 (70.7%)	6,612 (72.9%)
**Exposure (SARS-CoV-2 infection)**			
**Gestation at first infection within exposure period**			
Pre-conception	645 (21.3%)	-	-
2+0-5+6 weeks	552 (18.2%)	-	-
6+0-10+6 weeks	589 (19.5%)	-	-
11+0-15+6 weeks	566 (18.7%)	-	-
16+0-19+6 weeks	673 (22.2%)	-	-
**Number of infections***			
1	3,014 (99.6%)	-	-
2+	11 (0.4%)	-	-
**Outcome (miscarriage)**			
**Pregnancies ending in miscarriage up to 19+6 weeks**	204	600	584

**Gestation at miscarriage**			
<=10+6	135 (66.2%)	536 (89.3%)	377 (64.6%)
11+0-13+6	44 (21.6%)	39 (6.5%)	139 (23.8%)
>=14+0	25 (12.3%)	25 (4.2%)	68 (11.6%)
**Imputed gestation for miscarriage**			
Yes	103 (50.5%)	488 (81.3%)	265 (45.4%)
No – available from ANC booking	74 (36.3%)	29 (4.8%)	249 (42.6%)
No – available from end of pregnancy record	27 (13.2%)	83 (13.8%)	70 (12.0%)
**Lag between first infection (or gestational week of matching in uninfected) and miscarriage**			
<2 weeks	21 (10.3%)	81 (13.5%)	90 (15.4%)
2-5 weeks	66 (32.4%)	159 (26.5%)	166 (28.4%)
6-9 weeks	53 (26%)	176 (29.3%)	162 (27.7%)
>=10 weeks	64 (31.4%)	184 (30.7%)	166 (28.4%)

SIMD=Scottish Index of Multiple Deprivation

*Between 6 weeks pre-conception and the earliest of either: end of pregnancy or 19+6 weeks gestation

All groups exclude women who received COVID-19 vaccination between 6 weeks pre-conception and the earliest of either: end of pregnancy or the end of the exposure period at 19+6 weeks gestation

Supplementary Table 5: Key characteristics of infected and control groups included in the infection and ectopic pregnancy analyses

	Infected cohort	Uninfected historical controls	Uninfected contemporary controls
**Key characteristics**			
**Number of pregnancies**	915	2,745	2,745
**Median age (min-max)**	28 (13-45)	28 (14-45)	28 (13-46)
**Deprivation (SIMD quintile)**			
1 (most deprived)	294 (32.1%)	718 (26.2%)	768 (28.0%)
2	223 (24.4%)	572 (20.8%)	607 (22.1%)
3	147 (16.1%)	481 (17.5%)	460 (16.8%)
4	148 (16.2%)	525 (19.1%)	527 (19.2%)
5 (least deprived)	91 (9.9%)	414 (15.1%)	362 (13.2%)
Unknown	12 (1.3%)	35 (1.3%)	21 (0.8%)
**Ethnicity**			
White	774 (84.6%)	1,765 (64.3%)	2,328 (84.8%)
South Asian	26 (2.8%)	47 (1.7%)	72 (2.6%)
Black/Caribbean /African	9 (1.0%)	28 (1.0%)	39 (1.4%)
Other/mixed ethnicity	23 (2.5%)	49 (1.8%)	69 (2.5%)
Unknown	83 (9.1%)	856 (31.2%)	237 (8.6%)
**Urban/rural status**			
Large urban areas	374 (40.9%)	970 (35.3%)	980 (35.7%)
Other urban areas	341 (37.3%)	1,036 (37.7%)	1,003 (36.5%)
Accessible small towns	59 (6.4%)	231 (8.4%)	233 (8.5%)
Remote small towns	15 (1.6%)	88 (3.2%)	85 (3.1%)
Accessible rural areas	49 (5.4%)	273 (9.9%)	285 (10.4%)
Remote rural areas	17 (1.9%)	107 (3.9%)	111 (4.0%)
Unknown	60 (6.6%)	40 (1.5%)	48 (1.7%)
**Clinical vulnerability**			
Extremely vulnerable	5 (0.5%)	30 (1.1%)	26 (0.9%)
Vulnerable	244 (26.7%)	762 (27.8%)	728 (26.5%)
Not vulnerable	666 (72.8%)	1,953 (71.1%)	1,991 (72.5%)
**Exposure (SARS-CoV-2 infection)***			
**Gestation at infection within exposure period***			
Preconception	769 (84.0%)	-	-
2 weeks (2+0 to 2+6)	146 (16.0%)	-	-
**Outcome (ectopic pregnancy)**			
**Pregnancies ending in ectopic pregnancy up to 19+6 weeks**	8	33	32
**Gestation at ectopic pregnancy**			
<=10+6	8 (100%)	33 (100%)	32 (100%)
**Imputed gestation for ectopic pregnancy**			
Yes	8 (100%)	30 (90.9%)	29 (90.6%)
No – available from ANC booking	0	0	3 (9.4%)

No – available from end of pregnancy record	0	3 (9.1%)	0
**Lag between infection (or gestational week of matching in uninfected) and ectopic pregnancy**			
<=5 weeks	0	1 (3.0%)	0
6-9 weeks	5 (62.5%)	18 (54.5%)	19 (59.4%)
>=10 weeks	3 (37.5%)	14 (42.4%)	13 (40.6%)

SIMD=Scottish Index of Multiple Deprivation
*It was only possible to have one infection in the exposure period (i.e. between 6 weeks pre-conception and 2+6 weeks gestation)
**Between 6 weeks pre-conception and the earliest of either: end of pregnancy or 2+6 weeks gestation
All groups exclude women who received COVID-19 vaccination between 6 weeks pre-conception and the earliest of either: end of pregnancy or the end of the exposure period at 2+6 weeks gestation

Supplementary Table 6: Additional information on potential confounders included in analyses

Variable	Categorisation	Notes
Deprivation: Scottish Index of Multiple Deprivation (SIMD)	Quintile from 1 (most deprived) to 5 (least deprived)<br>Unknown	SIMD is a relative measure of deprivation calculated for each of 6,976 areas across Scotland. It draws on data on income, employment, education, health, access to services, crime and housing.[1,2]
Ethnicity	White<br>South Asian<br>Black/Caribbean/African<br>Other/mixed ethnicity<br>Unknown	This classification is based on standard categories used in the Scottish decennial population census.[3]
Rural urban status	Large urban areas<br>Other urban areas<br>Accessible small towns<br>Remote small towns<br>Accessible rural areas<br>Remote rural areas<br>Unknown	We have grouped this according to the standard Scottish Government urban-rural categorisation.[4] Where possible rural urban information was derived from postcode as identified from: general acute hospital discharge records (Scottish Morbidity Record (SMR) 01); maternity hospital discharge records (SMR 02); statutory termination of pregnancy notifications; the National Records of Scotland (NRS) statutory live birth registrations and the NRS statutory stillbirth registrations; where postcode was not available from these data sources it was taken from GP records (if available). As we rely on data from women's maternity records and other sources capturing the end of pregnancy, we have higher levels of missing for this covariate among ongoing pregnancies in the COPS dataset.
Clinical vulnerability	Clinically extremely vulnerable (on highest risk list)<br>Clinically vulnerable (but excluding diabetes)<br>Not clinically vulnerable	Individuals considered clinically extremely vulnerable to SARS-CoV-2 infection were identified from the national highest risk/shielding list maintained by Public Health Scotland,[5] with data kept up-to-date at the time the COPS cohort dataset was created for this study (26 April, 2022). Individuals considered clinically vulnerable were those not on the highest risk list whose primary care/GP record included a diagnostic code indicating they were in any Q-COVID risk group (excluding diabetes) or had hypertension.[6] These data are based on what was reported to GPs, with cross-sectional data available in June 2020 and January 2021. The data available in June 2020 was used to assign pregnancies between January 2015 and December 2020 to clinical vulnerability categories, with all

		later pregnancies assigned to categories based on the more recent cross section of data.<br>We intended to include diabetes as a separate confounder variable, based on information recorded in women's maternity records, however this was not feasible as information was missing for a high proportion of women with early pregnancy loss.

[1]Scottish Government. Scottish Index of Multiple Deprivation 2020 (Accessed 16 June 2022 at: https://www.gov.scot/collections/scottish-index-of-multiple-deprivation-2020/)
[2]Public Health Scotland. GPD Support: Deprivation (Accessed 16 June 2022 at: https://www.isdscotland.org/Products-and-Services/GPD-Support/Deprivation/SIMD/)
[3]IDS Scotland. Data Dictionary A-Z: Ethnic Group (Accessed 16 June 2022 at: https://www.ndc.scot.nhs.uk/Dictionary-A-Z/Definitions/index.asp?Search=E&ID=243&Title=Ethnic%20Group)
[4]Scottish Government. Scottish Government Urban Rural Classification 2016. (Accessed 16 June 2022 at: https://www.gov.scot/publications/scottish-government-urban-rural-classification-2016/)
[5]Public Health Scotland. COVID-19 – Search criteria for highest risk patients for inclusion to the shielding list (Accessed 22nd September 2022 at: https://hpspubsrepo.blob.core.windows.net/hps-website/nss/3008/documents/1_covid-19-search-criteria-highest-risk-patients.pdf)
[6]Oxford University. QCovid® risk calculation (Accessed 16 June 2022 at: https://www.qcovid.org/)

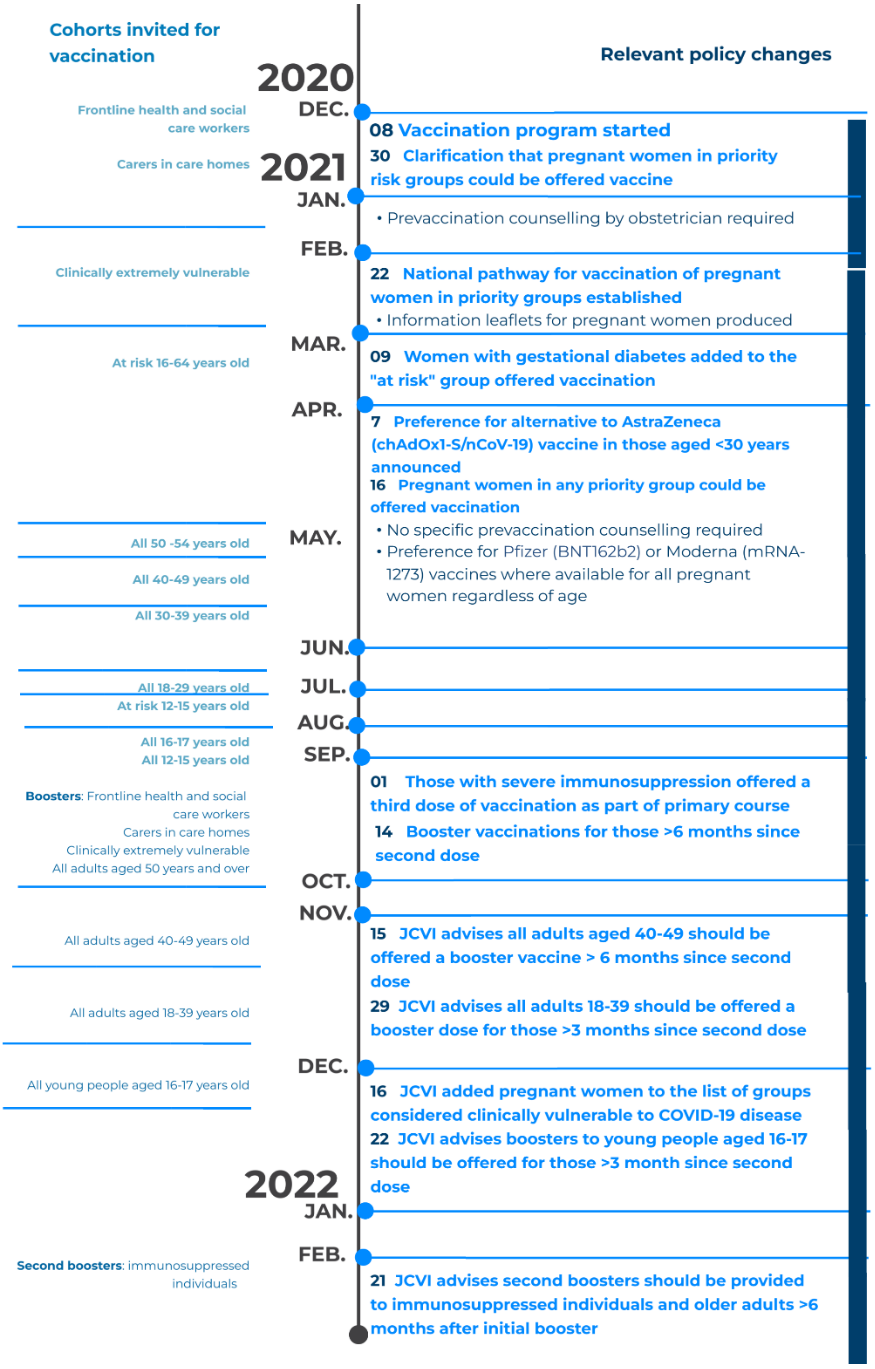

Supplementary Figure 1: ***Timeline of the COVID-19 vaccination programme in Scotland as relevant to pregnant women***

Summary of population cohorts invited for COVID-19 vaccination over time in Scotland from the start of the vaccination program on 8 December 2020 (left) and policy changes relevant to vaccination in pregnant women (right).

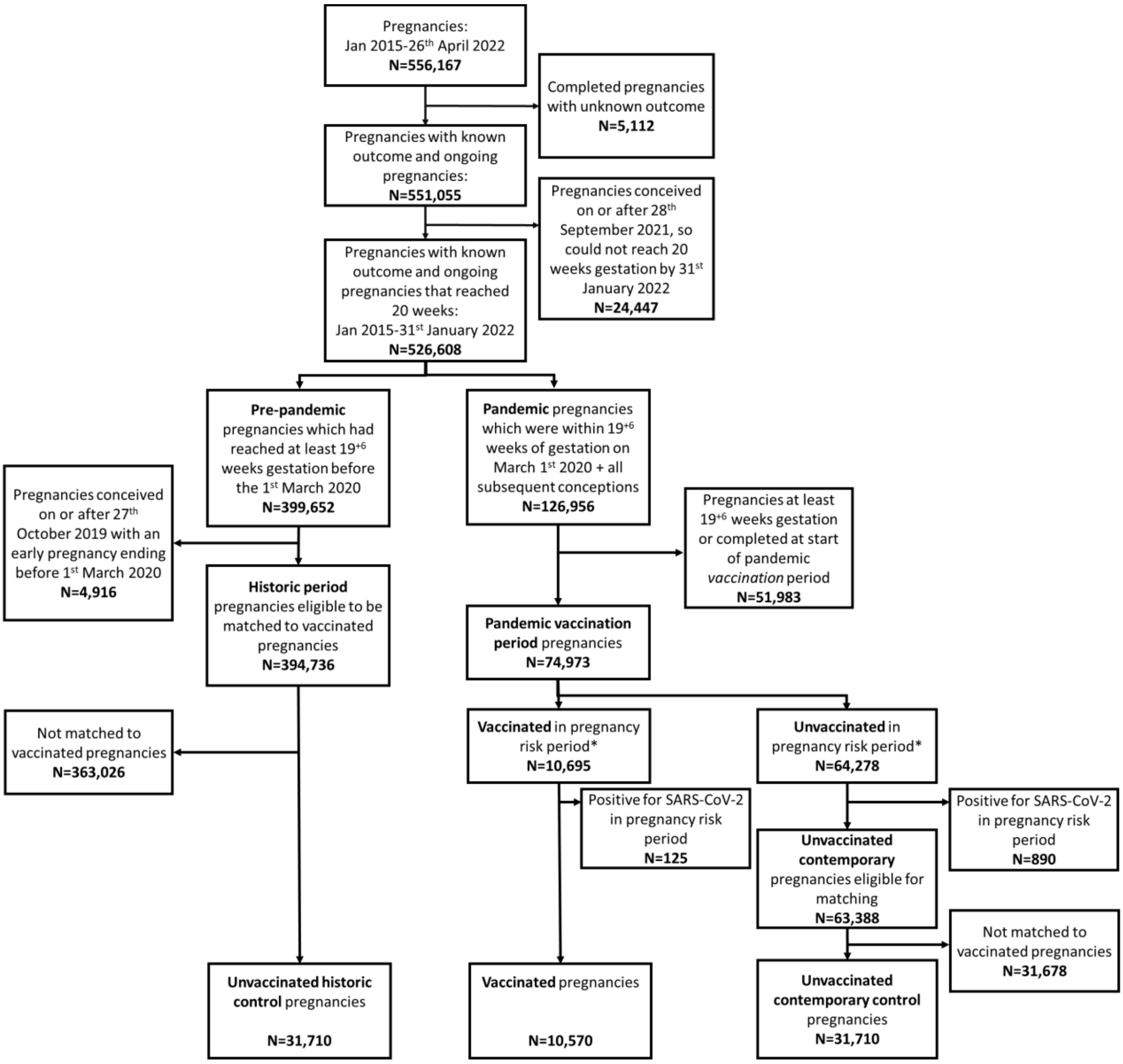

Supplementary Figure 2. ***Selection of vaccinated and unvaccinated pregnancy cohorts for ectopic outcome analysis***

*Flow diagram showing the selection of pregnancies for the analysis of the association between COVID-19 vaccination and risk of ectopic pregnancy. *For ectopic analysis, vaccination needs to be given between six weeks preconception and up to the earliest of: (1) end of pregnancy or (2) two weeks and six days gestation.*

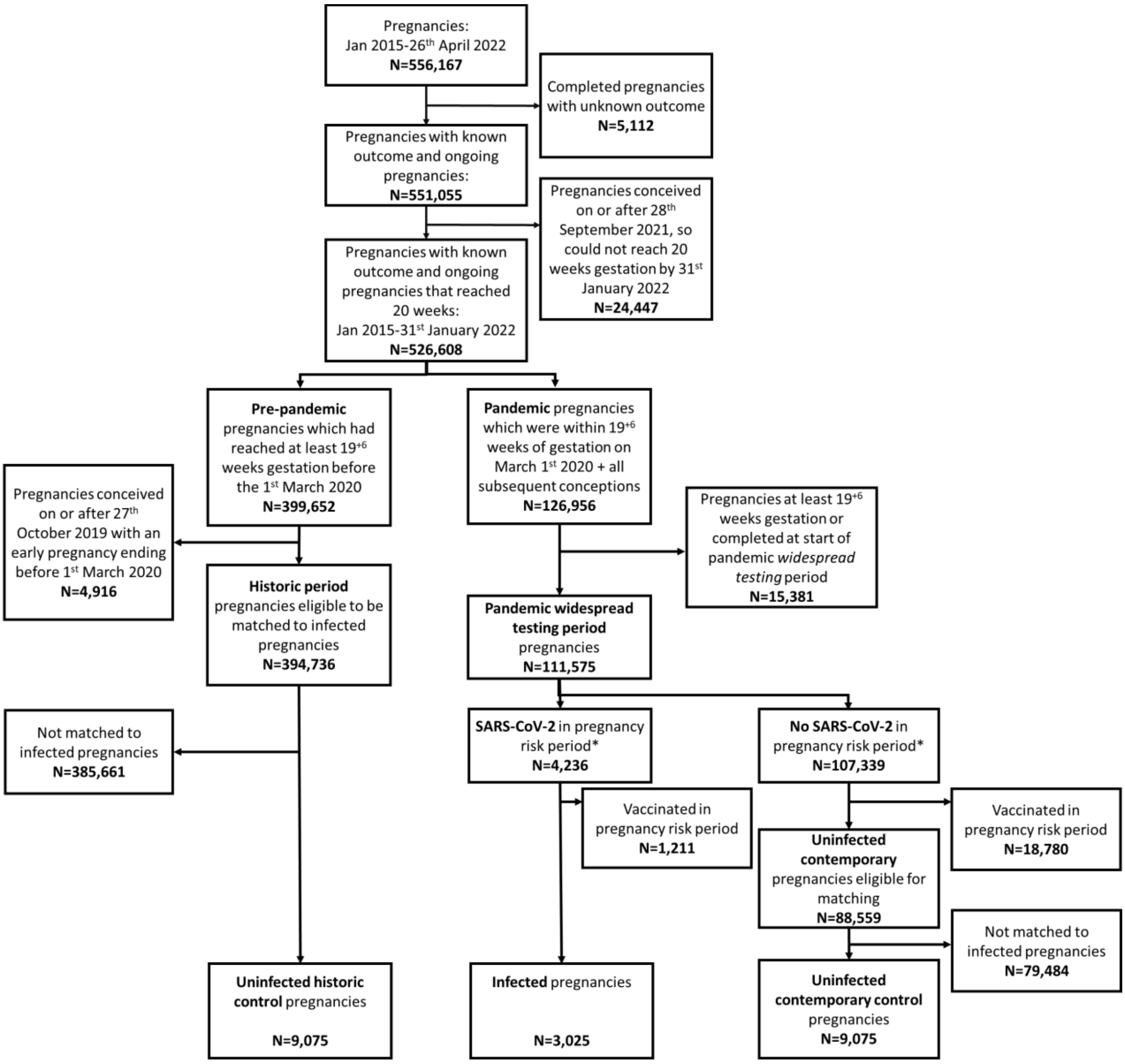

Supplementary Figure 3. ***Selection of infected and uninfected pregnancy cohorts for miscarriage outcome analysis***

*Flow diagram showing the selection of pregnancies for the analysis of the association between SARS-CoV-2 infection and risk of miscarriage. *For miscarriage analysis, SARS-CoV-2 infection needs to be recorded between six weeks pre-conception and up to the earliest of: (1) end of pregnancy or (2) 19 weeks and six days gestation.*

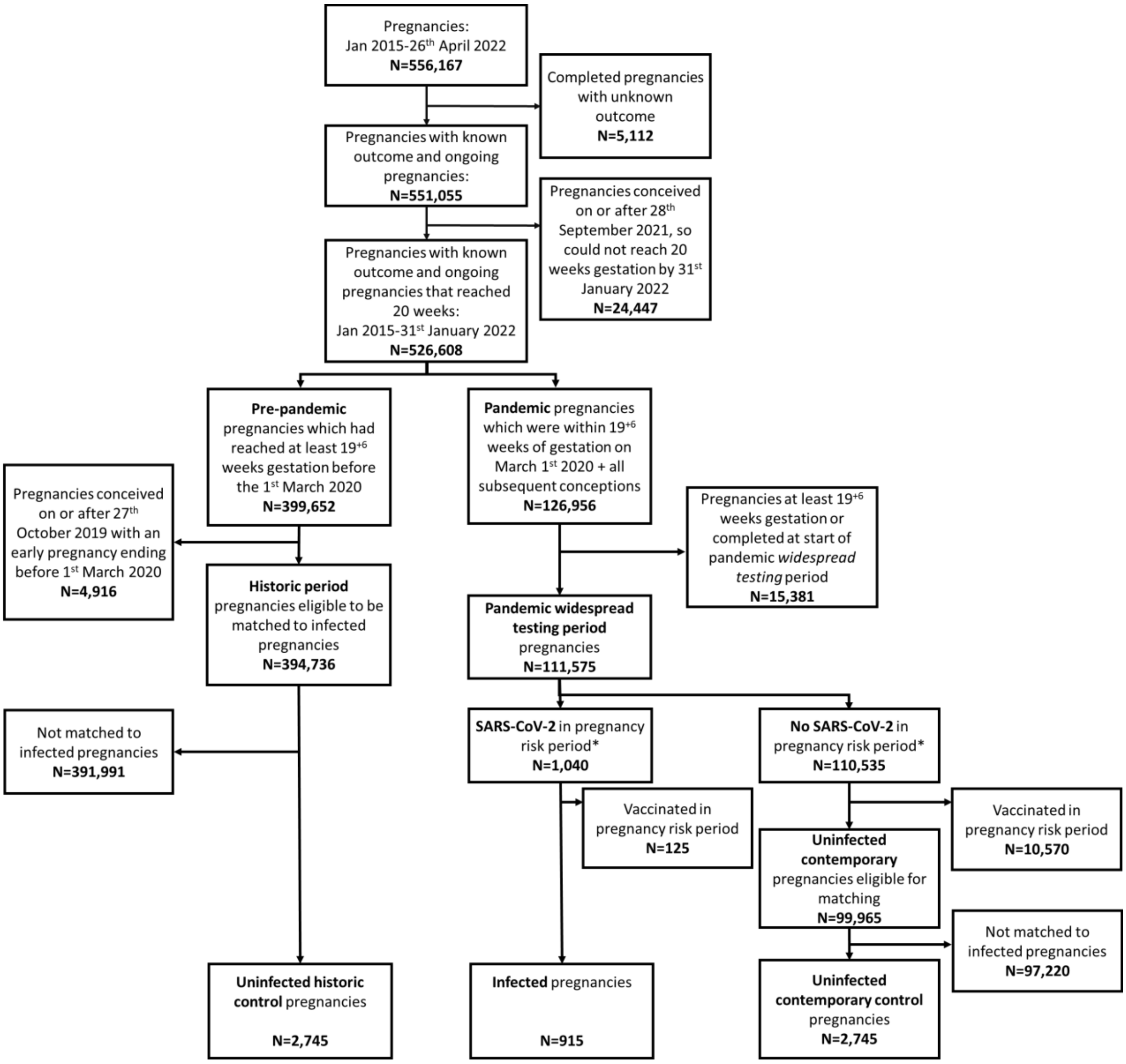

Supplementary Figure 4. ***Selection of infected and uninfected pregnancy cohorts for ectopic pregnancy outcome analysis***

*Flow diagram showing the selection of pregnancies for the analysis of the association between SARS-CoV-2 infection and risk of ectopic pregnancy. *For ectopic pregnancy analysis, SARS-CoV-2 infection needs to be recorded between six weeks pre-conception and up to the earliest of: (1) end of pregnancy or (2) 2 weeks 6 days gestation.*
